# Spatial analysis of colorectal cancer outcomes: investigating the impact of place-specific factors using causal inference methods for spatial data

**DOI:** 10.1093/pubmed/fdaf044

**Published:** 2025-05-07

**Authors:** Dajana Draganic, Knut R Wangen

**Affiliations:** Department of Health Management and Health Economics, Institute of Health and Society, University of Oslo, Forskningsveien 3A, Oslo 0373, Norway; Department of Health Management and Health Economics, Institute of Health and Society, University of Oslo, Forskningsveien 3A, Oslo 0373, Norway

**Keywords:** causal methods for spatial data, colorectal cancer, place-specific factors, regional disparities, screening rates

## Abstract

**Background:**

Disparities in health outcomes across regions may arise from place-specific factors, encompassing both contextual elements such as healthcare accessibility and compositional factors tied to the unique population characteristics. This study seeks to investigate the impact of various place-specific factors on late-stage incidence and mortality rates within Norwegian municipalities.

**Method:**

Municipality-level data on colorectal cancer (CRC) late-stage diagnosis and mortality rates were acquired from the Cancer Registry of Norway and the Norwegian Cause of Death Registry. Screening utilization rates were obtained from the Norwegian Patient Registry. To explore the region-level effects of place-specific factors on CRC outcomes, a causal inference method for spatial data—neighborhood adjustment method via spatial smoothing (NA approach)—was employed.

**Results:**

The findings indicate that a one-unit increase in screening rates (or a 1% rise in screening uptake) corresponds to a 2.9% decrease in late-stage incidence, with a 95% credible interval ranging from −0.055 to −0.003. However, no significant relationship between screening rates and mortality rates was observed.

**Conclusion:**

This study underscores the importance of maximizing the utilization of screening services to prevent advance-stage diagnosis. Moreover, the research underscores the significance of improving access to screening services, particularly in rural and medically underserved areas.

## Introduction

Geographical disparities in colorectal cancer (CRC) incidence are prevalent across numerous countries, attributable to various factors and exposures. Within Norway itself, CRC incidence varies significantly, with differences exceeding 20 per 100 000 person-years between regions characterized by high and low incidence rates.^1^^,^^2^ Disparities may stem from place-specific factors, in addition to patient-related factors such as one region having a higher concentration of sicker individuals than another. While patient-related factors may not be a significant concern for policymakers, understanding the impact of place-specific factors on health outcomes is crucial for addressing geographic disparities and identifying inefficiencies.^3^

Existing research suggests that countries with higher colorectal screening uptakes generally exhibit better health outcomes including lower colorectal (CRC) incidence and mortality rates.^4–8^ These studies which are mostly randomized control trials and observational studies, have demonstrated the efficacy of commonly employed screening tests, such as fecal tests, sigmoidoscopy, and colonoscopy in reducing incidence and mortality rates. However, significant regional and sub-population disparities in screening rates and CRC outcomes persist, with noticeable spatial patterns.^2^^,^^9^ Understanding the population-level impact of differently designed screening programs on regional disparities, remains an area of limited exploration with challenges to establish a direct causal link.^4^^,^^10^

Other place-specific factors that could contribute to disparities in CRC outcomes through inadequate access to preventive services and healthy lifestyles could be divided into contextual and compositional factors.^11^ Compositional factors linked to CRC outcome disparities are connected to demographics (age, sex, and race/ethnicity), socio-economic status and health behaviors (rates of smoking, physical activity, diet, and similar). Low socioeconomic status (SES) counties are linked to higher CRC mortality rates.^12–14^ Additionally, populations characterized by specific racial and ethnic backgrounds, as well as those residing in rural areas, exhibit higher incidence and mortality rates.^15–17^ Contextual factors are related to healthcare accessibility and quality, built environment (urban vs. rural settings), difference in culture and environmental exposures. In the context of our study, we use physician density (general practitioner and specialist density) as proxies for gaging healthcare accessibility. Physician density is usually associated with increased screening uptake for CRC, and thus with better outcomes.^18–21^

While numerous studies underscore the significance of place-specific factors in cancer outcome regional disparities, few consider the spatial interdependencies between different regions. Some geographers and economists emphasize the spatial position of an area, pointing to similar screening uptakes in neighboring districts due to concepts of informal communication and observational learning.^21^^,^^22^ Beyond local interactions leading to spatial dependencies, place-specific drivers can cluster due to various reasons.^23^ Namely, spatial interdependence can occur because local healthcare providers are part of a single regional authority, which influences the quality and quantity of healthcare services available. Additionally, other factors, such as climate, pollution, and crime, are more strongly linked to regional trends than to local ones, thereby exhibiting some form of spatial dependence. Addressing this gap, our study emphasizes the importance of accounting for these spatial interdependences and spillover effects when analyzing regional variations in cancer outcomes by applying spatial models.

Our study represents the first study to explore the impact of place-specific factors on incidence and mortality rates through the application of causal inference methods for spatial data. Acknowledging the limitation of traditional spatial approaches, particularly in establishing causal interpretations, our approach seeks to enhance the existing literature. By employing novel causal methods for spatial data, our study aims to contribute to a more nuanced understanding of the correlation between health service utilization and regional incidence and mortality rates, effectively accounting for spatially linked unobserved variables arising from various regional and spatial interdependencies.^24^ Moreover, the spatial effect holds potential for guiding local policy planning and healthcare resource allocation, facilitating the development of targeted, spatially focused programs to reduce health inequalities.

## Methods

### Data and measures

This study examined variations in CRC outcomes across all 356 municipalities in Norway, which is the lowest administrative level (division from 2020). The study focused on CRC late-stage incidence and mortality rates between 2016 and 2021, obtained from the Cancer Registry of Norway and Norwegian Cause of Death Registry. The dataset consisted of the total number of CRC cases and total number of deaths. It encompassed the annual counts of newly diagnosed late-stage CRC cases and deaths at the municipal level. The primary outcome variable of interest was the standardized incidence and mortality ratio (SMR), computed from the collected data as the ratio between observed and expected incidence and mortality cases. The expected number of cases was determined by applying late-stage incidence and mortality rates specific to the age and gender groups of the whole Norwegian population to the observed area. The age-sex groups were divided into four strata divided on females and males and population aged 0–64 and 65 years and older.

The main exposure variable, the utilization rates of screening services were calculated using data obtained from the Norwegian Patient Registry (NPR). This dataset included the number of screening procedures undertaken by patients during 2016 and related to specific screening procedures such as sigmoidoscopy. The data were collected at municipality levels and from these records, we computed utilization rates as the number of patients who used the service at least once within a year, divided by the number of the eligible population.

In addition, our investigation aimed at examining other place-specific factors associated with variations in CRC outcomes, including municipality-level socio-economic parameters encompassing socio-economic covariates measures, degree of rurality and proximal factors of the availability of healthcare services such as density of primary care practitioners (PCPs) and specialists related to CRC screening services (Table 1). The PCP and specialist densities were computed as the number of physicians per 1000 residents. Data relating to rurality and physician density were sourced from Statistics Norway, while information on socioeconomic covariates was provided by the Norwegian Institute for Public Health (Norwegian Institute of Public Health, 2024).

**Table 1:** Descriptive statistics for outcome- and independent variables.

Variable name	Description	Median (Q1–Q3)
Late-stage incidence rate^a^	Ratio between observed and expected late-stage incidence cases	1.10 (0.92–1.30)
Mortality rate^a^	Ration between expected and observed mortality cases	1.10 (0.81–1.30)
Screening rates	Percentage of population that has undertaken screening procedure (sigmoidoscopy)	0.0013 (0.0010–0.0021)
PCP density^b^^,^^c^	Number of primary care physicians per 1000 population	0.99 (0.84–1.20)
Specialist density^c^^,^^d^	Number of specialists related to CRC screenings per 1000 population	0.47 (0.20–1.30)
Rurality^b^	Percentage of area with rural settlements	0.47 (0.26– 0.67)
Elderly inhabitants^b^^,^^c^	Percentage of population over 65 years old	0.18 (0.15–0.20)
Income^e^	Median income (×1000)	390 (370–450)
Immigrants^e^	Percentage of population with no citizenship	0.09 (0.07–0.11)

^a^Outcome variables. ^b^Statistics Norway. ^c^Authors’ calculations. ^d^Cancer Registry of Norway (“Gastronet” project). ^e^Norwegian Institute for Public Health (Municipal Health Statistics Bank).

### Statistical analysis

We analyzed the correlation between CRC incidence, mortality rates, and screening rates at the municipal level while accounting for spatial dependencies. Additionally, we examined the influence of socioeconomic factors, rurality, immigration status, and healthcare service availability on CRC outcomes.

To assess spatial patterns and autocorrelation, we applied Global Moran’s I statistics as a diagnostic tool and aiming to provide insights into the overall spatial autocorrelation within dataset. The main analysis consisted of comparing two modeling approaches: a classical spatial model for count data and a neighborhood adjustment (NA) model via spatial smoothing. The NA model is a causal inference approach for spatial data designed to account for unobserved spatial confounders that could bias traditional spatial estimators (see Supplementary File 1 for details).

For the purpose of Global Moran’s I statistics and these two statistical methods, we first constructed a contiguity matrix (W) in the initial step. This matrix determined the connectivity between municipalities, indicating whether two municipalities shared a border (assigned a weight of 1) or did not (assigned a weight of 0).

Both models were implemented within a Bayesian framework to estimate the impact of screening rates on late-stage incidence and mortality, using Poisson-distributed outcomes. Municipality-level rates were calculated as the ratio of observed to expected cases, adjusted for national age- and gender-specific rates. We included covariates (Table 1) and assumed Gaussian priors with a mean of 0 and a standard deviation of 10.

A total of 356 municipalities were analyzed. Prior research suggests that Bayesian spatial models remain effective even with low spatial autocorrelation or a limited number of regions, provided there are at least 25 spatial units.^25^ All statistical analyses were conducted in R, with model codes adapted from Schnell and Papadogeorgou’s GitHub repository (https://github.com/schnellp/causal-spatial).

Further methodological details, including the justification of the method and the estimation process of the NA estimator, are provided in the Supplementary File 1.

## Results

### Descriptive statistics

Table 2 is showing the late-stage CRC incidence and mortality counts and rates per 10 000 population for sex, and age. From 2016 to 2021, there were a total number of 4652 death attributed to CRC, resulting in an annual mortality rate of 17.9 deaths per 10 000 population. In relation to late-stage incidence there were 7737 new cases with the rate of 29.8 cases per 10 000 population. For both cancer outcomes, males aged 65 and above exhibited higher mortality rates than women, while for the age below 65 the difference between males and females is less pronounced.

**Table 2:** CRC late-stage incidence and mortality counts by sex and age, 2016–2021.

	Mortality				Late-stage incidence			
Age groups	Female		Male		Female		Male	
	Count	Rate	Count	Rate	Count	Rate	Count	Rate
Total	4652	17.9	4726	18.0	7737	29.8	8300	32.1
<65	810	3.8	1009	4.5	2004	9.4	2449	10.9
over 65	3842	82.6	3717	95.3	5733	123.3	5851	150.0

**Figure 1:**
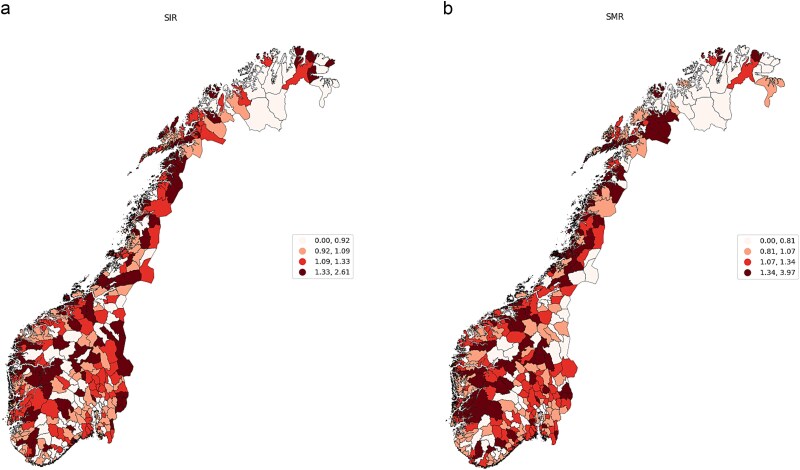
(a) Municipality-level standardized late-stage incidence rate for CRC, 2016–2020, Norway. (b) Municipality-level standardized mortality rates for CRC, 2016–2020, Norway.

Figure 1a and b shows the distribution of the standardized incidence and mortality rates (adjusted by age and sex) across all Norwegian municipalities. The figure indicates a notable concentration of similar rates in adjacent regions.

The observation of the clustered incidence and mortality rates is further supported by the Global Moran's I test for overall spatial correlation. Spatial correlation is significant and positive, with mean rate of 0.15 for late-stage incidence rates and 0.14 for standardized mortality rates and *P*-value ˂0.05. The positive value is indicating the presence of both high and low incidence and mortality rates being clustered together geographically.

### Statistical analysis results

Table 3 presents findings from both spatial and NA models for place-specific factors influencing two CRC outcomes: late-stage incidence and mortality rates. Although the spatial model indicates no significant relationship, the NA model demonstrates a significant correlation between screening rates and the late-stage incidence rate. According to NA model results, the mean coefficient (−0.029) suggests that a one-unit increase in screening rates from sigmoidoscopy (or a 1% increase in screening uptake) corresponds to a 2.9% decrease in late-stage incidence, with a 95% credible interval between −0.055 and −0.003. However, according to this model, no significant relationship between screening rates and mortality rates is evident based on the credible interval. Moving on to other place-specific factors, there is a notable correlation between rurality and primary care physician (PCP) density with late-stage incidence rates. A one-unit increase in PCP density (or an increase of one physician per 1000 inhabitants) is associated with an 8.7% decrease in late-stage CRC, while higher rurality is linked to a 4.3% increase in late-stage CRC (CI 0.005–0.081). In relation to the immigration factor, a one percentage point rise in the immigrant population correlates with a 6.3% reduction in late-stage incidence, with a significant credible interval (CI –0.094 to −0.031). Conversely, no significant relationship is observed between the specialist density and the two outcomes, as per the credible interval. Additionally, there is no evidence of a significant correlation with socio-economic status.

**Table 3:** Percentage change in the CRC incidence and mortality rates given the infinitesimal change in the exposure.

Late-stage incidence rate		
	Spatial model	NA model
Screening rates	−0.027 (−0.079 to 0.023)	−0.029 (−0.055 to −0.003)
Rurality	0.027 (−0.030 to 0.082)	0.043 (0.005 to 0.081)
PCP density	−0.078 (−0.143 to −0015)	−0.087 (−0.137 to −0.037)
Specialist density	−0.018 (−0.100 to 0.057)	−0.005 (−0.079 to 0.063)
Income	−0.025 (−0.074 to 0.023)	−0.018 (−0.045 to 0.009)
Immigrants	−0.051 (−0.102 to −0.001)	−0.063 (−0.094 to −0.031)
*Mortality rate*		
	*Spatial model*	*NA model*
Screening rates	0.012 (−0.051 to 0.073)	−0.018 (−0.051 to 0.014)
Rurality	0.049 (−0.062 to 0.069)	0.022 (−0.025 to 0.067)
PCP density	−0.051 (−0.131 to 0.027)	−0.054 (−0.19 to 0.007)
Specialist density	−0.034 (−0.136 to 0.059)	−0.027 (−0.118 to 0.059)
Income	−0.031 (−0.085 to 0.024)	−0.033 (−0.068 to 0.002)
Immigrants	−0.044 (−0.102 to 0.011)	−0.051 (−0.089 to −0.012)

Concerning mortality rates, only immigrants exhibit a significant correlation, according to the NA model, indicating a 5.1% decrease in mortality with a one percentage increase in immigrant population. Rurality, physician density, income and screening rates do not demonstrate a significant relationship with mortality rates based on the credible interval.

**Figure 2:**
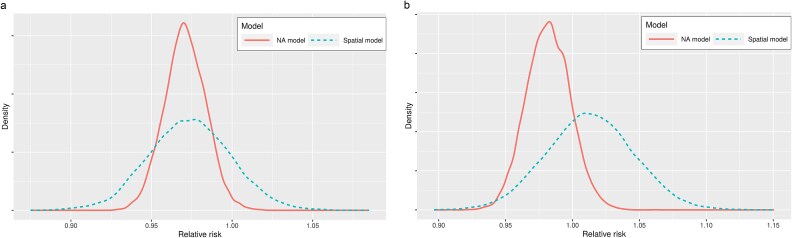
(a) Posterior density distribution of municipality level relative risk of late-stage incidence rate and screening rates. (b) Posterior density distribution of municipality level relative risk of mortality from CRC and screening rates.

Figure 2a and b depict the posterior distribution of the exponentiated relative risk for both the spatial and NA models (population average response curve), representing the relative expected risk of late-stage incidence and mortality rates with a one percentage point increase in screening rates in a randomly chosen municipality. The results in Fig. 2a indicate that the posterior mean of the relative risk for screening rates was ˂1, specifically equal to 0.97 (CI 0.94–0.99) according to the NA method. This suggests a protective effect of higher screening rates, implying that increased screening rates lead to a lower expected relative risk of late-stage incidence. However, when compared with the spatial model, the credible interval was considerably wider, ranging between 0.91 and 1.02, indicating non-significant results.

Figure 2a and b presents the posterior distributions of the exponentiated relative risk for both models, representing the relative expected risk of mortality with a one percentage point decrease in screening rates. Based on the credible interval results, both spatial and NA models show non-significant results.

## Discussion and conclusion

### Main findings of the study

The present study is the first study to evaluate regional disparities in CRC outcomes, specifically late-stage incidence, and mortality, by using causal inference method for spatial data. These findings reinforce outcomes from previous studies, particularly in contrast to spatial studies that have not been sufficient to identify a relationship between screening rates and CRC outcomes.^10^ Contrary, our study reveals that areas with higher screening rates, tends to have lower-than-expected late-stage incidence rate. Our study highlights the screening rate as a key factor contributing to disparities in CRC outcomes, as demonstrated by the results from the NA model. Additionally, our study highlights the impact of primary care physician (PCP) density and rurality on CRC outcome disparities. An increase in PCP density is linked to both decreased late-stage incidence and mortality, while rural areas tend to exhibit higher-than-expected incidence rates.

The most important finding from our study is that higher screening rates are associated with lower-than-expected late-stage incidence rates, emphasizing the crucial role of screening in preventing advanced-stage diagnoses. The entirely negative 95% credible interval (−5.5% to −0.3%) supports a protective effect of screening. While the lower bound suggests a modest impact, the upper bound indicates a significant reduction in late-stage incidence. However, the cumulative effect across all municipalities could still be clinically significant, even at the lower bound. Also, small reductions in late-stage cancer incidence can have significant impact by improving survival rates, enhancing quality of life, and lowering healthcare costs. In terms of mortality, no relationship between screening rates and mortality was found, possibly due to our relatively short follow-up period.

### What is already known on this topic

Based on our study results, the PCP density is significantly influencing the incidence of late-stage CRC, aligning with previous research findings.^18^^,^^20^^,^^26^ These findings highlight the important role of primary care physicians in cancer screening. However, the density of specialists did not emerge as a significant factor in reducing CRC incidence and mortality rates. While some prior studies suggest that specialist density impacts cancer outcomes,^20^ others argue that the balance between primary and specialist physician supply is inconsequential.^27^ Consistent with earlier studies, our findings also indicate that rural areas exhibit higher-than-anticipated rates of late-stage CRC incidence.^15^^,^^28^ Multiple factors may contribute to this observation, including socioeconomic differences between rural and urban areas or travel distances. Our study did not discern any correlation between income and CRC outcomes, implying that travel distances to screening services may be a prominent factor in Norwegian contexts.

Our study has yielded negative findings regarding the association between immigration and CRC outcomes. This aligns with previous research conducted in Norway and several European countries indicating that lifestyle-related cancers like CRC exhibit lower incidence and mortality rates among immigrant populations compared to the native population.^29–31^ These observations can be attributed to the “healthy migrant effect” or differences in lifestyle choices, given that CRC incidence is closely linked to Western dietary and lifestyle patterns. However, despite immigrants displaying lower CRC incidence rates overall, a German study indicates that late-stage cancer diagnoses are prevalent among non-Western female immigrants.^32^ Nonetheless, due to limitations in available data, we were unable to investigate variations among different immigrant groups, different generations of immigration or to distinguish between the impacts of lifestyle factors and healthcare utilization on outcome.

### What this study adds

This study has several strengths. It considers population-level impact of screening programs across the whole country and gives insights into regional disparities and how important they can be in designing screening programs. Notably, this study is the first to evaluate the impact of cancer screening rates on CRC outcomes using causal inference methods for spatial data such as NA model, strengthening causal conclusions compared to standard spatial models. This was also evidenced by robustness checks on the key assumption of positivity.

The spatial methodology employed in this study holds the potential for replication in diverse geographic settings, when examining place-specific factors influencing CRC outcomes. This methodology enables spatial smoothing across regions by leveraging data from neighboring areas, which is especially beneficial for Norwegian municipalities with limited cancer case reports.

The study underscores the importance of spatial monitoring in addressing health inequalities in Norway and advocates for the reduction of these disparities through targeted spatial strategies and adaptable program designs. Launched on 25 August 2022, Norway’s National Colorectal Cancer Screening Program currently offers biennial home-taken FIT testing for ages 55–65, with plans to transition to a one-time colonoscopy at 55 as resources allow. However, this shift must consider potential access inequalities, especially for those living farther from screening centers. Specifically, interventions and policies should be directed toward areas with poor accessibility to screening services and specifically within rural populations.

### Limitations of this study

Nevertheless, the study is subject to limitations as it relies on aggregated data, precluding individual-level inferences. For example, it does not account for other factors related to the decision-making process for screening, which are influenced by individual-level factors such as personal beliefs, awareness, and access to information. However, despite this constraint, the results obtained at the municipality level still provide valuable insights for policymakers, shedding light on local and regional contextual factors that influence screening utilization and outcomes. The spatial focus of the study underscores its potential as an alternative to observational studies and clinical trials in shaping targeted policy programs for reducing health inequalities. However, drawing causal conclusions from this approach requires assuming that the spatial scale of the confounder is larger than that of the exposure, meaning smaller-scale confounders may not be accounted for. One limitation of this study is the temporal misalignment between screening rates and incidence/mortality rates, which fails to capture the full extent of the screening impact. This discrepancy may underestimate the effect, as the effects of screening rates are unlikely to manifest fully within the short time frame covered by this study.

In conclusion, our study emphasizes that increased screening rates play significant role in reducing late-stage incidence, underscoring the key importance of the better utilization of screening services to prevent advanced-stage diagnoses. Since the study period predates Norway's first national organized screening program and its transition to one-time colonoscopy, future research should evaluate its impact, particularly in rural and underserved areas.

## Supplementary Material

supplement_file_1_fdaf044

## Data Availability

Raw data were generated from Cancer Registry of Norway and Norwegian Patient Registry. Derived data supporting the findings of this study are available from the corresponding author DD on request. The data are not publicly available due to restrictions e.g. their containing information that could compromise the privacy of research participants.

## Notes

**Dajana Draganic,** Doctoral Research Fellow

**Knut R. Wangen,** Associate professor
