# Supplementary material for: Spatial analysis of colorectal cancer outcomes: investigating the impact of place-specific factors using causal inference methods for spatial data
Source: J Public Health (Oxf). 2025 May 7;47(3):446–53. doi: 10.1093/pubmed/fdaf044 (PMC12395940; doi:10.1093/pubmed/fdaf044)
Supplement: supplement_file_1_fdaf044 [file supplement_file_1_fdaf044.docx]

**SUPPLEMENT FILE 1**

**Additional information on neighbourhood adjustment via spatial smoothing methodology**

In our analysis we adopted the neighbourhood adjustment method approach, which is a part of broader spatial causal inference methods, to account for spatial correlation and alleviate bias arising from spatially correlated unobserved confounders. Namly, the motivation behind these methods lies in the fact that spatial data often violate the assumptions underpinning standard causal inference techniques, requiring novel theories and computational tools.^1^

Similar to non-spatial regression, spatial regression also encounters difficulties in accurately estimating the effects of explanatory variables when unmeasured common causes are present. These unmeasured factors can influence both the dependent variable (outcome) and the explanatory variables or create interdependencies among the explanatory variables themselves. In spatial analysis, this phenomenon is referred to as a spatial confounder—an unobserved variable or factor that varies geographically and simultaneously affects the outcome and one or more independent variables. This spatial confounder frequently exhibits spatial patterns, and their omission can lead to biased estimates.

Since health outcomes are often influenced by shared, difficult-to-measure or unmeasured environmental, social, or policy factors, these models effectively address these spatial correlations that exists across regions.

Motivated by the limitations of commonly used spatial estimators, the authors of this method introduced a novel estimator, the affine estimator, which includes a component that depends on omitted spatial variables.^2^

Namely, the authors of the model argue that the commonly used approach to estimate $\hat{\beta}$ is biased when $u_{i}$ is present as this bias arises from a non-zero correlation between the confounder and exposure, resulting in a non-zero conditional expectation E($u_{i}$|Z). ^2^ The equation is as follows:

$Y_{i} = {\beta Z}_{i} - B(X) + \gamma C_{i}+\varepsilon_{i}$ (1)

where $B(X)=$ E($u_{i}$|X) is the bias term and X is the design matrix containing the intercept, exposure, and measured covariates.

Given the infeasibility of estimating the bias term E($u_{i}$|X) through traditional methods, we evaluated the plausibility of assumptions proposed by authors, that encompass both spatial and causal aspects of the affine estimator. This set of assumptions comprises standard causal assumptions, structural assumptions, and additional assumptions essential for estimating the causal exposure-response curve derivative using the affine estimator.

The casual assumptions in our study include standard causal inference assumptions, including temporal ordering and SUTVA (Stable Unit Treatment Value Assumption), along with an additional one known as the positivity assumption. This positivity assumption refers to spatial scale restrictions of unmeasured confounders, suggesting that estimating the causal effect of exposure on the outcome is viable only if the variability in exposure aligns with the levels of the confounder or if the spatial scale of the confounder exceeds that of the exposure.

To ensure the temporal ordering of exposure and outcome, we selected the screening rate dataset from 2016, while the incidence and mortality data were derived from a dataset spanning the years 2016 to 2021. We assert the plausibility of the Stable Unit Treatment Value Assumption (SUTVA) since we postulate that the impact of screening uptakes at the municipality level on incidence and mortality rates is primarily driven by individual-level causal effects, influencing only individuals within their respective municipalities. This assumption is corroborated by the fact that individuals diagnosed with or deceased from colorectal cancer (CRC) between 2016 and 2020 are presumed to have resided in their respective municipalities during that timeframe. In section 3.2, we validated the robustness of our model by assessing the positivity assumption—specifically, by comparing the affine estimator with and without spatial scale restriction of the unmeasured confounder (unconstrained and constrained NA model).

Structural assumptions include outcome additivity, Cross-Markov property, and constant conditional correlation. Outcome additivity assumes that the exposure and measured covariates do not interact with the unmeasured covariates and can be partially validated with standard diagnostic. Cross-Markov property and constant conditional correlation are assumptions about the relationship between unobserved confounder and the exposure to which standard diagnostic is not applicable.

We estimated the plausibility of the outcome additivity by visual examinations. Namely, a scatterplot of the joint distribution of the residual log exposure after adjusting for covariates versus mean imputed confounder U appeared Gaussian. The credibility of Cross-Markov property and constant conditional correlation is validated based on specific context and the hypothetical confounding factors under consideration. For example, if we consider an unmeasured variable such as physician practice style, this variable can act as a confounder as it can affect both, the screening rates, and the outcomes. The cross-Markov property allows for physician practice style to exhibit distinct relationships across different locations. However, it is assumed that within a given municipality, this variable exclusively impacts screening rates within that municipality and do not directly affect neighbouring municipalities and their respective screening rates. Other potential confounders in the analysis, beyond variations in physician practice styles, include public health campaigns, healthcare infrastructure, socioeconomic status, cultural beliefs, social norms, and other related factors that could influence both screening rates and outcomes. The constant conditional correlation implies that the strength of the relationship between physician practice style and screening rates are constant across municipalities.

In this study, the positivity assumption, which is crucial for causal interpretation, was assessed by comparing the results of the posterior distributions of both unconstrained and constrained NA models (models with and without spatial scale restrictions). The unconstrained NA model for late-stage incidence and screening rates showed a Gaussian distribution with a mean of 0.44 and a standard deviation of 0.67. The restricted NA model, which incorporates spatial scale limitations, yielded similar posterior distributions. The consistency between these two models suggests that the positivity assumption holds within the scope of the study. The assumption implies that estimating the causal effect of screening rates on incidence and mortality is feasible only if there is variability in exposure (screening rates) across levels of confounders. If the spatial scale of confounders is smaller than that of exposure, the positivity assumption would be violated, as certain "strata" of confounders may only allow one value of exposure.

Literature:

(1) Reich, B. J.; Yang, S.; Guan, Y.; Giffin, A. B.; Miller, M. J.; Rappold, A. A Review of Spatial Causal Inference Methods for Environmental and Epidemiological Applications. *International Statistical Review* **2021**, *89* (3), 605–634. https://doi.org/10.1111/insr.12452.

(2) Schnell, P. M.; Papadogeorgou, G. Mitigating Unobserved Spatial Confounding When Estimating the Effect of Supermarket Access on Cardiovascular Disease Deaths. *The Annals of Applied Statistics* **2020**, *14* (4), 2069–2095. https://doi.org/10.1214/20-AOAS1377.
